# Behavioral Context Determines Network State and Variability Dynamics in Monkey Motor Cortex

**DOI:** 10.3389/fncir.2018.00052

**Published:** 2018-07-12

**Authors:** Alexa Riehle, Thomas Brochier, Martin Nawrot, Sonja Grün

**Affiliations:** ^1^UMR7289 Institut de Neurosciences de la Timone (INT), Centre National de la Recherche Scientifique (CNRS)—Aix-Marseille Université (AMU), Marseille, France; ^2^Institute of Neuroscience and Medicine (INM-6), Institute for Advanced Simulation (IAS-6) and JARA Brain Institute I, Forschungszentrum Jülich, Jülich, Germany; ^3^Computational Systems Neuroscience, Institute for Zoology, University of Cologne, Cologne, Germany; ^4^RIKEN Brain Science Institute (BSI), Wako, Japan; ^5^Theoretical Systems Neurobiology, RWTH Aachen University, Aachen, Germany

**Keywords:** monkey motor cortex, spike time irregularity, spike count variability, behavioral context, renewal processes

## Abstract

Variability of spiking activity is ubiquitous throughout the brain but little is known about its contextual dependance. Trial-to-trial spike count variability, estimated by the Fano Factor (FF), and within-trial spike time irregularity, quantified by the coefficient of variation (CV), reflect variability on long and short time scales, respectively. We co-analyzed FF and the local coefficient of variation (CV2) in monkey motor cortex comparing two behavioral contexts, movement preparation (*wait*) and execution (*movement*). We find that the FF significantly decreases from *wait* to *movement*, while the CV2 increases. The more regular firing (expressed by a low CV2) during *wait* is related to an increased power of local field potential (LFP) beta oscillations and phase locking of spikes to these oscillations. In renewal processes, a widely used model for spiking activity under stationary input conditions, both measures are related as FF ≈ CV^2^. This expectation was met during *movement*, but not during *wait* where FF ≫ CV2^2^. Our interpretation is that during movement preparation, ongoing brain processes result in changing network states and thus in high trial-to-trial variability (expressed by a high FF). During movement execution, the network is recruited for performing the stereotyped motor task, resulting in reliable single neuron output. Our interpretation is in the light of recent computational models that generate non-stationary network conditions.

## Introduction

A number of *in vivo* studies have demonstrated a high variability of single neuron spiking activity in the neocortex. Here, we address the question whether and how cortical spiking statistics within a trial and across trials may depend on the behavioral context aiming at an improved understanding of the underlying nature and sources of neuronal variability. In our experimental analyses, we co-analyze two types of spiking statistics that reflect variability on separate time scales (Nawrot et al., 2008; Murray et al., 2014). *Spike time irregularity* (Perkel et al., 1967; Softky and Koch, 1993) refers to the random appearance of a sequence of spikes. This finds quantitative expression in the dispersion of inter-spike intervals (ISIs) within a trial, statistically captured by the *coefficient of variation* (CV) of ISIs. It mainly represents variability on a relatively short time scale, in the range of tens to a few hundreds of milliseconds, determined by the typical duration of ISIs (Softky and Koch, 1993; Holt et al., 1996; Nawrot et al., 2008). *Trial-by-trial spike count variability* measures the variation in the number of spikes across repeating experimental trials of the same behavioral condition. It can statistically be quantified by the *Fano factor* (FF; Shadlen and Newsome, 1998; Nawrot et al., 2008) defined as the ratio between the variance and the mean of spike counts measured across trials within an observation window of predefined length. It represents variability on a rather long time scale in the range of seconds determined by the typical separation of trials that belong to an identical behavioral condition. For both measures, high CV and FF values indicate irregular ISIs and high spike count variability, respectively.

Spike time irregularity is much lower in the sensory periphery (Werner and Mountcastle, 1963) or motor periphery (Calvin and Stevens, 1968; Clamman, 1969; Prut and Perlmutter, 2003; Barry et al., 2007; Duclos et al., 2008) than in cortical primary sensory and motor areas. Within cortical areas, the CV decreases systematically from visual to higher-order sensorimotor areas (Maimon and Assad, 2009; Shinomoto et al., 2009; Mochizuki et al., 2016). Likewise, the FF differs with stages of sensory processing, being lowest in the periphery and highest in the cerebral cortex (Kara et al., 2000).

Other studies have explored the modulations of spike time irregularity and spike count variability in relation to behavior. Davies et al. (2006) showed that in motor cortex spike time irregularity increases during the most demanding epoch of a precision grip task. Spike count variability, on the other hand, systematically decreases during a behavioral task being lowest during movement execution (Churchland et al., 2006, 2010; Rickert et al., 2009), perceptual processing (Mitchell et al., 2009; Churchland et al., 2010; Abolafia et al., 2013; Ponce-Alvarez et al., 2013; Mazzucato et al., 2015), attention (Mitchell et al., 2007; but see McAdams and Maunsell, 1999), or in relation to decision processes (Churchland et al., 2011). In addition, Rickert et al. (2009) showed that during movement preparation and execution the FF in motor cortex depends on the amount of prior information about the requested movement. However, these studies suffer important limitations. First, they do not systematically assess how changes in spiking variability relate to changes in firing rate. Second, these studies analyzed spike time irregularity and spike count variability separately although *in vitro* studies suggest that these two measures may be directly related (Nawrot et al., 2008).

Here, we take advantage of a very large database of single neuron recordings from the motor cortex of three macaque monkeys to decipher the relationship between firing rate, spike time irregularity and spike count variability. In particular, we demonstrate how this relationship changes in different behavioral contexts, specifically during movement preparation and execution.

## Materials and Methods

### Behavioral Task

Three adult macaque monkeys (*Macaca mulatta*), two females (monkey L and E), weighing 4.5 and 7 kg, and one male (monkey N), weighing 7 kg, were used in the experiment. All animal procedures were approved by the local ethical committee (“Comité d’Ethique en Neurosciences INT Marseille,” registered at the “Comité National de Réflexion Ethique sur l’Expérimentation Animale” under the number 71-C2EA 71; authorization A1/10/12) and conformed to the European and French government regulations.

Details of the task and recording procedures were described in Riehle et al. (2013), Milekovic et al. (2015) and Brochier et al. (2018). Monkeys were trained to perform an instructed delay reach-to-grasp task to obtain a food reward (apple sauce), using the left hand. They sat in a custom-made primate chair in front of the experimental apparatus with the non-working (right) arm loosely restrained in a semi-flexed position. The unrestrained working hand rested on a switch positioned at waist-level, 5 cm lateral to the midline. The target object was a stainless steel, rectangular parallelepiped (40 × 16 × 10 mm) attached to the anterior end of a low-friction horizontal shuttle and rotated at a 45° angle from the vertical axis. It was located 13 cm away from the switch at 14 cm height. The object had to be grasped and pulled with the working hand using one of two different grip types: a precision grip (PG) or a side grip (SG). The object weight could be set to one of two different values (100 or 200 g) by means of an electromagnet inside the apparatus. Thus, the force required to pull the object was either low force (LF) or high force (HF). Changes in object weight occurred between trials and were undetectable by the monkey. The apparatus provided a continuous measure of the grip and pulling (load) forces by means of force sensitive resistances (FSR). In addition, a hall-effect sensor measured the horizontal displacement of the object over a maximal distance of 15 mm.

A square of four red light-emitting diodes (LEDs) with one additional yellow LED in its center was used to display the instruction cues. The LEDs were inserted in the apparatus just above the target object. Illumination of the two left or right red LEDs instructed the monkey to perform a SG or a PG, respectively. Illumination of the two bottom or top LEDs instructed the monkey that pulling the object required a LF or HF, respectively.

**Figure 1:**
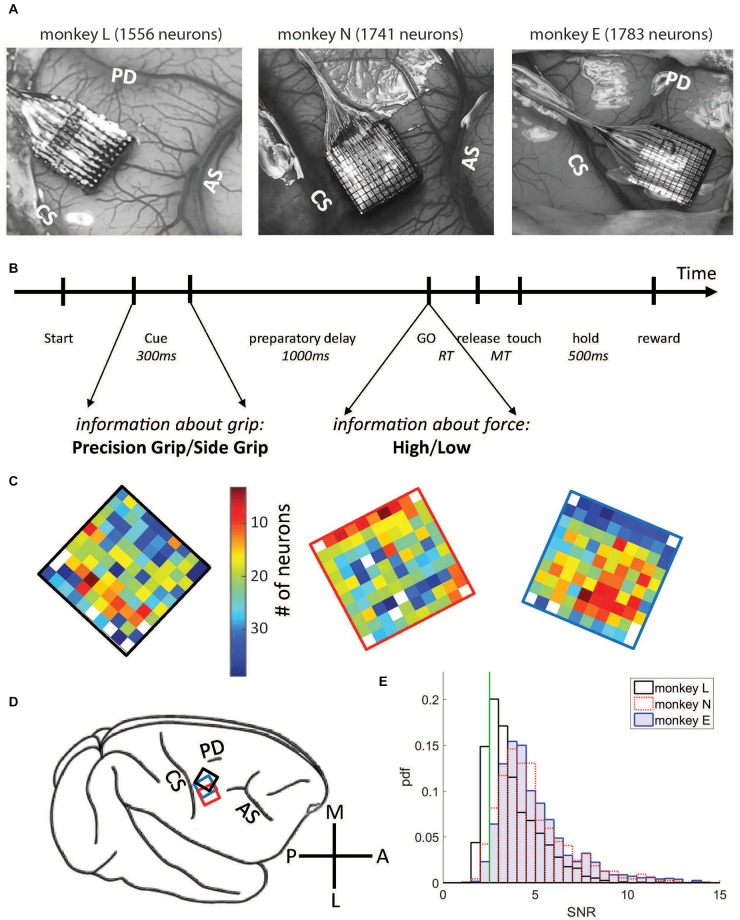
Data set and electrode array locations. **(A)** Locations on the cortical surface of the Utah arrays implanted in monkey L (left), N (middle) and E (right), all in the right hemisphere (see **D**). Pictures were taken during surgery. CS, central sulcus; AS, arcuate sulcus; PD, precentral dimple. **(B)** Time line of the task. **(C)** Numbers of selected neurons on each electrode, signal-to-noise ratio (SNR) >2.5. Same color code (# of neurons) for both monkeys (monkey L at the left with a black frame, monkey N at the middle with a red frame, and monkey E at the right with a blue frame). **(D)** A schematic drawing of the right hemisphere including the placement of the arrays for each monkey using the same colors as for the frames shown in **(B)**. M, medial; A, anterior; L, lateral; P, posterior. **(E)** Distributions of SNR values for all neurons for all three monkeys. Vertical green line corresponds to the selected SNR threshold value (2.5).

The task was programmed and controlled using LabView (National Instruments Corporation, Austin, TX, USA). The trial sequence was as follows (see Figure 1B). The monkey had to close the switch with the hand to self-initiate a trial (trial start, TS). After 400 ms, the central yellow LED was illuminated for another 400 ms (warning signal, WS), followed by the preparatory cue (Cue), illuminated for 300 ms, which instructed the monkey about the grip (PG or SG) required to perform the trial. Cue extinction was followed by a 1 s preparatory delay. At the end of this delay, the GO signal provided the remaining information about the force and also served as the imperative signal asking the monkey to release the switch (switch release; SR) and to reach and grasp the object. Following object grasp, the monkey had to pull the object towards him/her into a narrow position window (4–14 mm) and to hold it there for 500 ms to obtain the reward (Rew). In case of grip error, the trial was aborted and all four LEDs were flashed as a negative feed-back. The reaction time (RT) was defined as the time between the GO signal and SR, and the movement time (MT) as the time between SR and grip force onset as detected by the FSR by using a fixed threshold. The monkey was required to keep RT and MT below 700 ms, for monkeys L and N, and 1000 ms for monkey E to be rewarded. Five to 10 sessions of about 10–15 min each were recorded per day, up to five recording days per week. During each session, the four trial types (SG-LF, SG-HF, PG-LF, PG-HF) were presented at random with equal probability. Monkeys usually achieved a total of 100 to 140 successful trials per session. Only one session per recording day was selected for analysis to strongly reduce the probability to analyze the same neurons twice (see more details below).

### Surgery

When the monkey was fully trained in the task and obtained 85% correct trials, a 100-electrode Utah array (Blackrock Microsystems, Salt Lake City, UT, USA) was surgically implanted in the motor cortex contralateral to the working hand (for a schematic representation of array placements, see Figure 1D). The array had an arrangement of 10 × 10 Iridium Oxide electrodes, each of them 1.5 mm long, with an inter-electrode distance of 400 μm. The surgery was performed under deep general anesthesia using full aseptic procedures. Anesthesia was induced with 10 mg/kg i.m. ketamine and maintained with 2%–2.5% isoflurane in 40:60 O_2_-air. To prevent cortical swelling, 2 ml/kg of mannitol i.v. was slowly injected over a period of 10 min. A 20 × 20 mm craniotomy was performed over the motor cortex and the dura was incised and reflected. The array was positioned on the cortical surface 2–3 mm anterior to the central sulcus at the level of the spur of the arcuate sulcus (see Figure 1A). The array was inserted using a pneumatic inserter (Array Inserter, Blackrock Microsystems) and covered with a sheet of an artificial non-absorbable dura (Preclude, Gore-tex). The live dura was sutured back and covered with a piece of an artificial absorbable dura (Seamdura, Codman). The bone flap was put back at its original position and attached to the skull by means of a 4 × 40 mm strip of titanium (Bioplate, Codman). The array connector was fixed to the skull on the opposite side with titanium bone screws (Codman). The skin was sutured back over the bone flap and around the connector. The monkey received a full course of antibiotics and analgesics before returning to the home cage.

### Recordings

Neuronal data were recorded using the 128-channel Cerebus acquisition system (NSP, Blackrock Microsystems). The signal from each active electrode (96 out of the 100 electrodes were connected) was preprocessed by a head stage (monkey L: CerePort plug to Samtec adaptor, monkeys N and E: Patient cable, Blackrock Microsystems) with unity gain and then amplified with a gain of 5000 using the Front End Amplifier (Blackrock Microsystems). The raw signal was obtained with 30 kHz time resolution in a range of 0.3 Hz to 7.5 kHz. From this raw signal, two filter settings allowed us to obtain two different signals by using filters in two different frequency bands, the local field potential (LFP, low-pass filter at 250 Hz) and spiking activity (high-pass filter at 250 Hz). The LFPs were sampled at 1 kHz and saved on disk. On each channel, the experimenter set online a threshold for detection and extraction of potential spikes. All waveforms crossing the threshold were sampled at 30 kHz and snippets of 1.6 ms duration for monkey L and 1.3 ms for monkey N and E were saved for offline spike sorting. All behavioral data such as stimuli, switch release, force traces for thumb and index fingers and object displacement were also fed into the Cerebus, sampled at 1 kHz and stored for offline analysis. For more details and two representative data sets, see Brochier et al. (2018).

### Data Set, Selection Criteria and Analysis Windows

Single neurons were sorted offline from the online extracted waveforms by using the Plexon Offline Spike Sorter (version 3.3.3, Plexon Inc., Dallas, TX, USA). One particularity of the Cerebus acquisition system is that after threshold crossing to extract a potential spike, a dead time corresponding to the duration of the waveform window (1.3 and 1.6 ms, respectively, see above) does not allow to detect a new signal during this window. Spike clusters which were separated significantly from each other and with less than 1% of ISIs of durations of ≤2 ms, including the dead time of the system, were considered as single neurons. However, in some exceptional cases (<1%) neurons were selected as single neurons with more than 1% of very short ISIs. These neurons exhibited clear high frequency bursting activity with a few clear distinguishable spikes with reproducible spike shapes in a very short time window. These neurons were included in our analysis. For further selection, we calculated from the spike shapes of each neuron the signal-to-noise ratio (SNR), defined as the amplitude, i.e., the mean of the trough-to-peak voltage, divided by twice the standard deviation of the entire signal (Hatsopoulos et al., 2004). For this study we selected only single neurons with SNR values >2.5 to guarantee good sorting quality (see also Rouse and Schieber, 2016). The distributions of the SNR values of all neurons are shown in Figure 1E. For further analysis, spike times were down-sampled to 1 kHz.

For data analysis, we selected representative sessions from the entire recording period. The criteria for selection were (i) a large number of recorded neurons (64–110 neurons in monkey L, 92–167 neurons in monkey N, 121–165 neurons in monkey E), and (ii) a continuous behavior throughout the session without interruption between trials. From monkey L, 21 sessions were selected, recorded from Oct. 5, 2010 until April 15, 2011, including 1929 single neurons in total. Of those, 1556 neurons remained with SNR >2.5 and were included in the analysis. From monkey N, 13 sessions were selected, recorded from June until Nov. 2014, including 1826 single neurons, of those 1741 neurons with SNR >2.5 were selected for the subsequent analysis. And finally, from monkey E, 13 sessions were selected, recorded during Dec. 2016 and Jan. 2017, including 1828 neurons, of those 1783 neurons with SNR >2.5 were selected for the subsequent analysis. A small fraction of these neurons were likely to be the same across different sessions (Dickey et al., 2009). For the number of neurons selected on each electrode across all selected sessions, see Figure 1C. Note that for the study presented here simultaneity of the recorded spike data is not relevant, but it was relevant to achieve a large sample. The sessions selected in monkey L were: l101005-002, l101006-002, l101007-001, l101008-003, l101013-002, l101014-002, l101015-001, l1011108-001, l101110-003, l101111-002, l101126-002, l101202-001, l101209-001, l101216-002, l101220-002, l110208-001, l110209-001, l110404-001, l110408-002, l110411-001, l110415-002; in monkey N: i140613-001, i140616-001, i140617-001, i140627-001, i140701-001, i140702-001, i140703-001; i140704-001, i140718-001, i140721-002, i140725002, i140917-002, i141117-001, and in monkey E: e161209-001, e161212-002, e161213-001, e161214-001, e161215-001, e161216-001, e161219-002, e161220-001, e161222-002, e170105-002, e170106-001, e170109-001, e170110-004.

### Computing Variability Measures

**Figure 2:**
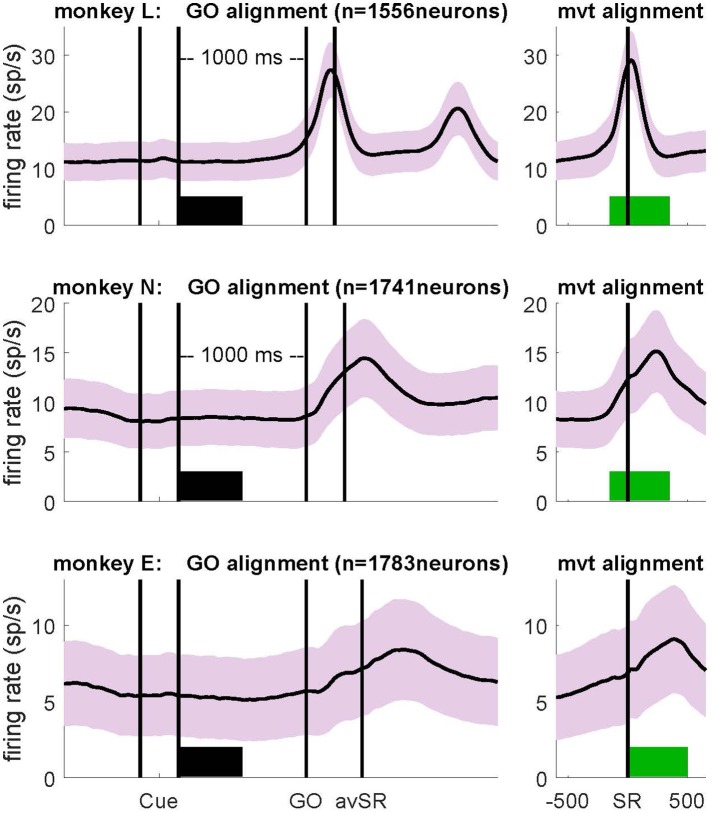
Firing rates averaged across all selected neurons (SNR > 2.5) during correct trials for monkey L (top), monkey N (middle) and monkey E (bottom), obtained during the trial type side grip-high force (SG-HF). Light colored envelopes indicate SEM. Data were aligned to the GO signal (left) and movement onset (SR; right). The selected analysis windows of 500 ms duration are indicated by a thick bar in black (*wait)* and green (*movement*). Cue: preparatory signal providing prior information about the grip type; GO: Go signal; SR: switch release (i.e., movement onset); avSR: average SR for data aligned to the GO signal. For firing rates recorded during each trial type as well as reaction times (RTs), see Supplementary Figure S1.

To analyze variability measures as a function of the behavioral context, we selected two discrete 500 ms epochs. To study neuronal activity during *wait*, data were aligned to the GO signal and the first 500 ms of the preparatory delay were selected, starting at Cue offset (horizontal thick black bars in Figure 2; left panels). We selected this specific window, because firing rate in most neurons was most stationary during this period. For the analysis of neuronal activity during *movement*, data were aligned to switch release (SR in the right panels of Figure 2, i.e., aligned to movement onset; in the left panels this event was indicated as avSR, meaning average SR times for data aligned to GO). In monkeys L and N a window from 150 ms before to 350 ms after SR was selected, whereas in monkey E a window from SR to 500 ms after SR was selected. These windows covered most of the movement period represented by the peak of average firing rate after GO (green horizontal bars in Figure 2, right panels). Note that monkey E had much longer RTs than the two other monkeys, see inset in Supplementary Figure S1.

The CV of ISIs is defined as

$$\text{CV}=\;\frac{SD\;(ISI)}{mean\;(ISI)\;}.$$

This measure is meaningful only for a constant firing rate and largely overestimates the irregularity of spiking activity if firing rate changes in time (see Ponce-Alvarez et al., 2010). To deal with non-stationary firing rates we used instead a local measure, the CV2 that was introduced by Holt et al. (1996). It is based on neighboring ISIs (*m*-values). Individual *m*-values were computed for any two consecutive ISIs as

$$m=2\frac{\left|\text{ISI}-\text{ISI′}\right|}{\text{ISI}+\text{ISI′}}$$

where *m* takes values between 0 (no variability) and 2 (maximum variability). Each *m*-value is associated with the time of occurrence of the second of the three considered spikes. For each analysis epoch (*wait* or *movement*), the CV2 is obtained by averaging across all *m*-values extracted in all trials that fall within the selected analysis window, where *N* is the number of *m*-values

$$CV2=\frac{1}{N}\sum_{i\;=\;1}^{N}m.$$

As a consequence, the 1st or the 3rd spike belonging to the two consecutive ISIs determining a respective *m*-value may be outside of the window. Reliability of the estimates requires a minimum of 20 *m*-values per window across all trials (see Ponce-Alvarez et al., 2010), thus segments with a lower number of *m*-values were not considered. We repeated the central analysis of our study using a different local measure of irregularity, the LV (Shinomoto et al., 2005), yielding the same qualitative results (see also Ponce-Alvarez et al., 2010).

The spike count variability was measured by the FF (Softky and Koch, 1993; Stevens and Zador, 1998) defined as

$$\text{FF}=\frac{var(n_{i})}{mean(n_{i})}$$

i.e., the ratio between the variance and the mean of the spike counts *n* across trials *i* within the analysis epoch (*wait* or *movement*). In order to minimize the estimation bias for the FF due to a finite window size we did not consider samples with less than 5 spikes/s per epoch. For processes that are more regular than Poisson, the bias then becomes negligible (Nawrot et al., 2008; Nawrot, 2010).

By theoretical argument, for any given stochastic point process the irregularity of ISIs (CV) and the variability of the spike count (FF) are related. Specifically for the model class of renewal processes, where ISIs are independent and equally distributed, the prediction is CV^2^ ≈ FF (Cox and Lewis, 1966; Perkel et al., 1967; Shadlen and Newsome, 1998; Chacron et al., 2001; Nawrot et al., 2008; Ditlevsen and Lansky, 2011; Farkhooi et al., 2011). In both measures, a Poisson process corresponds to a value of 1 and lower/higher values to lower/higher variable spiking activity. The prediction CV^2^ ≈ FF seems to be fulfilled *in vitro* (Stevens and Zador, 1998; Nawrot et al., 2008), but not *in vivo* as observed in the awake behaving monkey (Nawrot, 2010). In the latter stationarity across trials is not given, such that conclusions on the renewalty of the data cannot be drawn.

### Phase-Locking of Spikes to LFP Beta Oscillations

Phase-locking of spikes to LFP beta oscillations was analyzed in extended windows of 1000 ms duration. For *wait*, the window was selected from 500 ms before until 500 ms after cue offset, and for the *movement* epoch a window was selected from 400 ms before until 600 ms after movement onset. In order to exclude trivial signal correlations between the occurrence of spikes and the phase of the LFP oscillation induced by volume conductance effects (Katzner et al., 2009), we averaged trial by trial for each electrode channel the LFP signals recorded from all directly neighboring channels (between four and nine channels as a function of its location on the array). From this averaged LFP signal, the maximum frequency in the beta range was determined between 10 Hz and 45 Hz from the power spectrum during the enlarged *wait* window. This frequency was then used to filter the LFP signals throughout the entire length of the behavioral trial with a zero-phase bandpass filter (Matlab *filtfilt*, max. frequency ± 5 Hz, Butterworth, four poles). From this filtered signal, we calculated the instantaneous phase of the LFP obtained via a Hilbert transformation (Matlab *hilbert*; see Figure 6B). We then related for each neuron recorded on that electrode its spike times separately during each of the two analysis windows to the beta phase of the LFP (Matlab *angle*). In order to obtain the significance of a possible phase-locking of the spikes to the beta oscillation (*p* < 0.05), we used the Rayleigh test for non-uniformity of circular data in each window (CircStat Toolbox for Matlab, *circ_rtest*, Berens, 2009). For each analysis window, only neurons were taken into account having more than 30 spikes, making that the number of neurons varied between the two windows.

**Figure 3:**
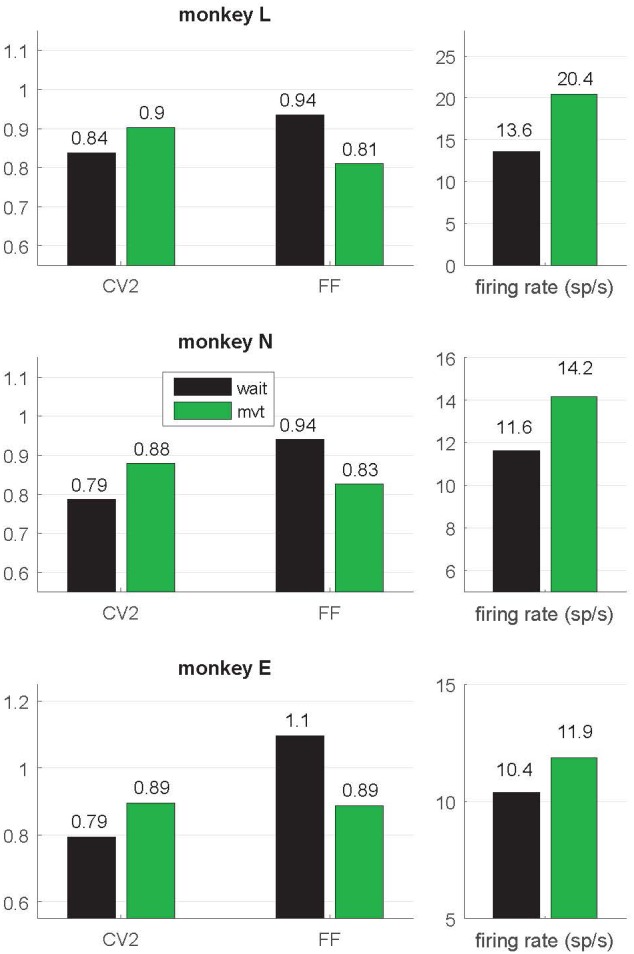
Context-dependent modulations of coefficient of variation (CV2), fano factor (FF) and firing rate during *wait* and *movement*. All values are indicated as medians and are significantly different (Wilcoxon rank sum test; *p* < 10^−4^) between *wait* (black) and *movement* (green) for all three monkeys. For the number of neurons, see Table 1. Data in this figure were obtained during the trial type SG-HF, values determined in all trial types are provided in Supplementary Table S1.

**Figure 4:**
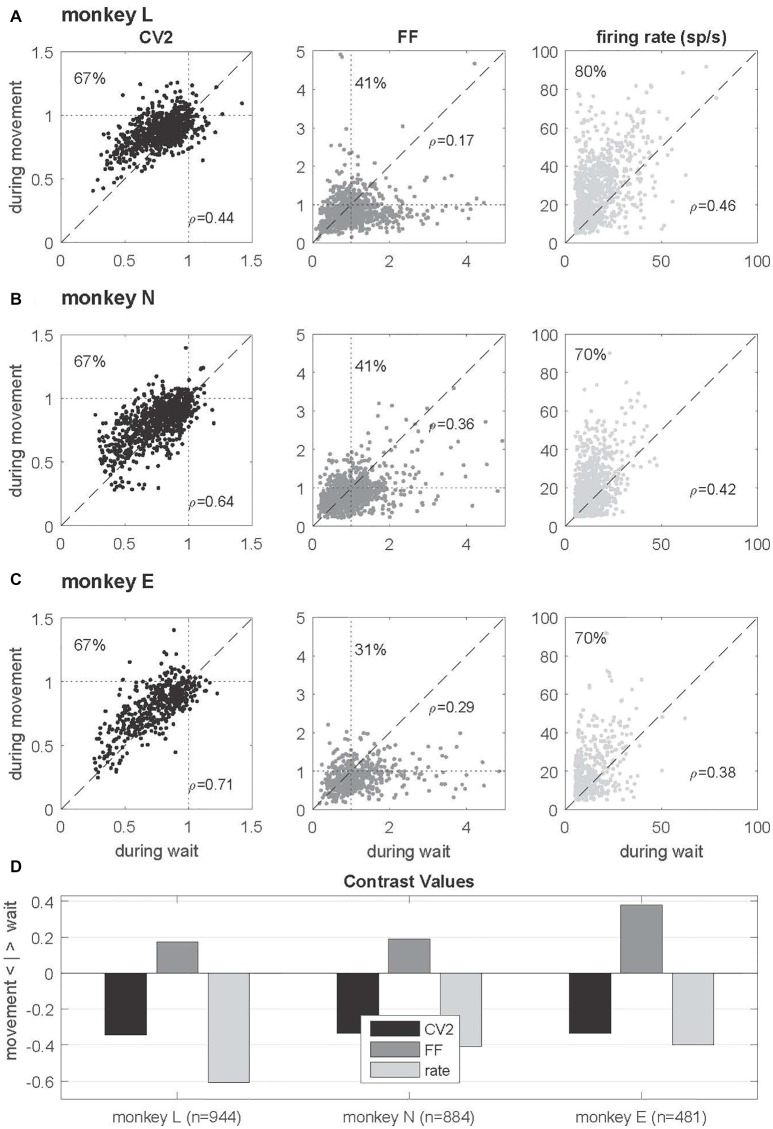
Single neuron comparison of variability measures and firing rate. **(A–C)** Scatterplots of CV2 (in black), FF (in dark gray) and firing rate (in light gray) obtained during *wait* (*x*-axis) and *movement* (*y*-axis) for monkey L (*n* = 944), N (*n* = 884) and E (*n* = 481) for neurons fulfilling the selection criteria in both epochs. The number in the upper left corner indicates the percentage of neurons whose values are higher during *movement* than during *wait*. In the right lower corner of each plot the rank correlation coefficient *rho* (Spearman) is indicated. All correlations are positive and highly significant (*p* < 0.001). **(D)** Contrast values for each feature. Data in this figure were obtained during the trial type SG-HF, for results in the other trial types see Supplementary Figure S2.

**Figure 5:**
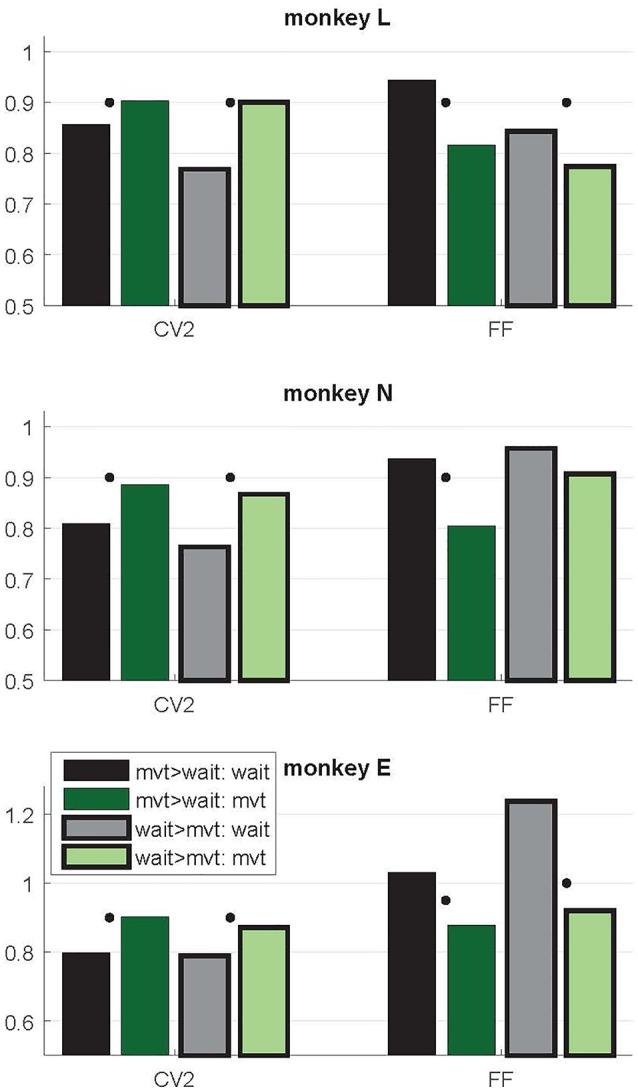
Context-dependent modulations of CV2 and FF during *wait* and *movement* as a function of the differential firing rate during the two task epochs. Data from trial type SG-HF. Variability measures obtained in two subpopulations of neurons, those with a higher firing rate during *movement* than during *wait* (mvt > wait; no surrounding, two left bars) and vice versa (wait > mvt; black surrounding, two right bars). For the numbers of neurons in each subpopulation, see Table 1. Medians of CV2 and FF during *wait* and *movement* for each subpopulation. For each measure and each subpopulation, the significant differences between *wait* and *movement* are indicated by a black dot (Wilcoxon rank sum test; *p* < 0.05). Data in this figure were obtained during the trial type SG-HF, for data obtained in all four trial types, see Supplementary Table S2.

**Figure 6:**
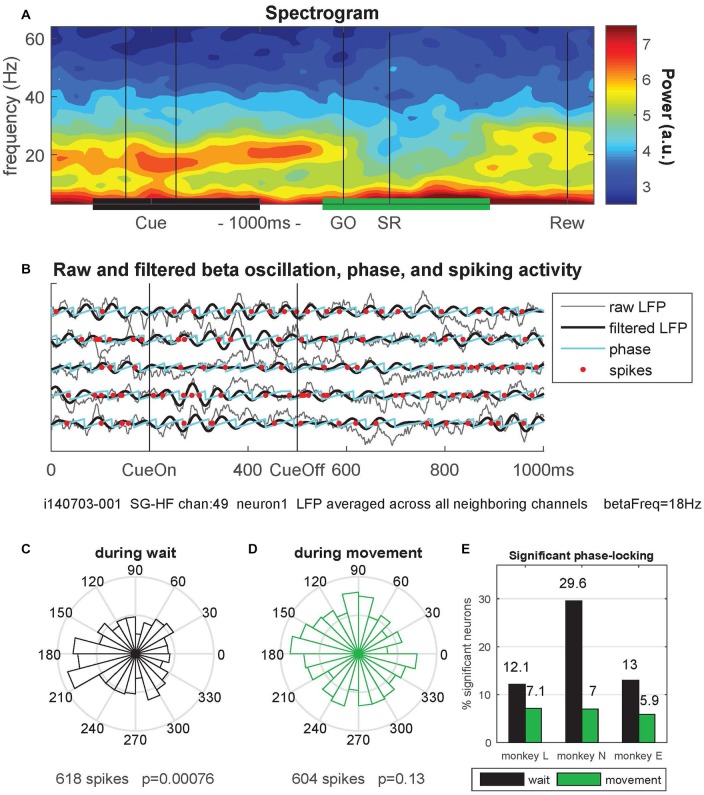
Phase-locking of spiking activity to local field potential (LFP) beta oscillations. **(A)** Spectrogram of the local field potential (LFP) signal averaged trial-by-trial across all neighboring electrodes around the reference electrode (channel 49) and then across all behavioral trials (*n* = 36) of a single trial type (here SG-HF) during one recording session in monkey N (i140703-001). The power is indicated as a color code in arbitrary units. The black and green horizontal bars at the bottom indicate the selected windows for calculating the phase-locking of spikes to the LFP oscillation phase. **(B)** The LFP and spiking data recorded on electrode 49 during five selected trials are shown, cut around cue presentation from 500 ms before until 500 ms after cue offset (CueOff). The duration of the cue was 300 ms. The raw (averaged) LFP signals are shown in gray. The signals in black were band-pass filtered around the main beta oscillation frequency (here 18 Hz ± 5 Hz; see “Materials and Methods” section). The oscillation phases obtained by Hilbert transformation are plotted in cyan. The spike times of one single neuron (neuron 1) recorded on channel 49 are indicated by red dots. **(C,D)** Circular representation of the phase relationships of the spiking activity of a single neuron obtained during *wait* **(C)** and during *movement* **(D)**, same neuron as the one presented in **(B)** by red dots, but recorded during all SG-HF trials. The number of spikes during the respective analysis window and the *p-value* of the outcome of the Rayleigh test are shown on the *x-axis* of the figures. **(E)** Percentages of statistically significant (*p* < 0.05) phase-locked neurons during *wait* (black) and *movement* (green) in the trial type SG-HF in monkeys L, N and E, respectively. For the number of selected neurons see Supplementary Figure S3 (upper panel). The results of the same analysis performed during all other trial types are shown in Supplementary Figure S3.

## Results

We explored in motor cortex the relationship between spike time irregularity and trial-by-trial spike count variability and analyzed the dependency of these two measures to changes in firing rate. For the spike time irregularity, we used instead of the classical CV of ISIs a local measure, the CV2, that was introduced by Holt et al. (1996) in order to deal with non-stationary firing rates. For the spike count variability, we determined the FF (see “Materials and Methods” section). Three monkeys (L, N and E) were trained in a delayed reach-to-grasp task (Riehle et al., 2013; Milekovic et al., 2015; Brochier et al., 2018) in which the animal had to grasp, pull and hold an object using either a SG or a PG and employing either a LF or HF, thus resulting in a total of four trial types (SG-LF, SG-HF, PG-LF, PG-HF). These trial types were presented to the animal in a pseudo-random fashion from trial to trial with equal probability in each recording session. We explored potential changes of the two variability measures, CV2 and FF, as a function of the behavioral context by selecting two contextually different epochs during the performance of the task. As a first epoch we chose a 500 ms-window directly after cue offset during movement preparation, subsequently called *wait*. During this epoch the monkey was not allowed to move but was requested to prepare the pre-cued movement based on the information provided by the cue. The second epoch was chosen during active movement execution, subsequently called *movement*. In each monkey, the 500 ms-window was roughly centered around the moment of the average peak discharge in the firing rate histogram of all neurons across all trials (see Figure 2). In monkeys L and N the 500 ms-window was chosen from 150 ms before to 350 ms after SR, whereas in monkey E who reacted more slowly to the GO signal, the 500 ms-window was chosen starting with SR (see Figure 2). We only considered single neuron activity (i) from trials of correct behavior, and (ii) with a high SNR (>2.5) of the spike shapes (see “Materials and Methods” section). Thus, the analysis in monkey L was performed on 1556 neurons (21 sessions), in monkey N on 1741 neurons (13 sessions), and in monkey E on 1783 neurons (13 sessions; Figure 1C, for the number of neurons on the electrode arrays). Figure 2 shows the trial-average firing rates of all selected neurons for each of the three monkeys during trials of one out of the four trial types (SG-HF).

For all our analyses, we aligned the neuronal activity to the GO signal (Figure 2, left) and to movement onset (i.e., SR; Figure 2, right) in the *wait* and the *movement* epoch, respectively. In the left panels of Figure 2 the two first vertical lines indicate the onset and offset of the preparatory cue, presented for 300 ms, that provided prior information about the grip type (here SG). The third vertical line indicates the occurrence of the GO signal that provided the missing information about the force to pull the object (here HF). The GO signal also requested the execution of the reach-to-grasp movement. Since the RT is variable across trials, SR times were averaged over all correct trials within each session, and indicated in Figure 2 (left panels) by a vertical line after GO as avSR (average switch release). The performance speed of the monkeys was different. Their average RTs across all trial types and sessions were 170 ms, 257 ms and 413 ms for monkey L, N and E, respectively. The second phasic rate increase visible in monkey L was due to her movements back to the center key, and is visible here only because her RTs were fast. For RTs obtained in each trial type for each monkey, see inset in Supplementary Figure S1.

We performed all analyses separately for each of the four trial types. However, since the results did not differ between them, we only show in the following the results for SG-HF, as in Figure 2. The results obtained during the other trial types are shown in the Supplementary Information.

### Irregularity (CV2) and Trial-By-Trial Variability (FF) Are Modulated With the Behavioral Context

In a first step of our analysis, we determined whether and how neuronal variability modulated with the behavioral context. For each single neuron we computed the spike time irregularity (CV2) and the spike count variability across trials (FF) separately in the two selected epochs *wait* and *movement*. For each epoch we considered only neurons that fulfilled our selection criteria for variability analysis, i.e., a minimum trial-averaged firing rate of 5 spikes/s and a sufficient number of ISIs to reliably compute CV2 and FF within the 500 ms window (see “Materials and Methods” section). All averages are expressed by the median. We find that both types of variability are modulated with the behavioral context. The CV2 is lower during *wait* than during *movement*, whereas the FF shows the opposite behavior (Figure 3). These contextual modulations are statistically highly significant (Wilcoxon ranksum test, *p* < 10^−4^) and are consistent across the three monkeys and across all four trial types (Supplementary Table S1). Additionally, the firing rate significantly increases from *wait* to *movement* (Figure 3 and Supplementary Table S1).

In order to explore the behavior of the individual neurons we analyzed the spiking activity of all neurons and selected the ones that fulfilled the selection criteria in both epochs. This drastically reduced the number of neurons (944, 884 and 481) in monkeys L, N and E, respectively (see Table 1). For those we inspected for each single neuron and each of the measures (CV2, FF and firing rate) its value observed during *wait* against that obtained during *movement* (Figures 4A–C). The obtained scatter diagrams show for each measure that in each monkey the majority of individual neurons indeed changed their behavior from *wait* to *movement*, consistent with the results gained from averaging across neurons (shown in Figure 3). In all three monkeys 67% of the neurons increased the CV2, whereas about 60%–70% of the neurons decreased the FF, and for a vast majority of neurons (70%–80%) the firing rate increased from *wait* to *movement*. This is summarized in Figure 4D by using the contrast value

$$C=\;\frac{n_{v_{wait}>v_{mvt}}-n_{v_{wait}<v_{mvt}}}{n_{v_{wait}>v_{mvt}}+n_{v_{wait}<v_{mvt}}}$$

where the two variables correspond to the number of neurons with a higher value during wait $n_{v_{wait}>v_{mvt}}$ and the other with a higher value during movement $n_{v_{wait}<v_{mvt}}$. This contrast measure signifies with a positive outcome a higher portion of higher values during *wait*, and vice versa, with a negative outcome a higher portion of higher values during *movement*.

**Table 1:** Selection of data sets, number of neurons (SG-HF).

	Monkey L	Monkey L	Monkey N	Monkey N	Monkey E	Monkey E
	*wait*	*mvt*	*wait*	*mvt*	*wait*	*mvt*
All recorded neurons/selected with SNR >2.5	1929/1556		1826/1741		1828/1783	
Selected neurons with >5 sp/s in either epoch (Figure 3)	981	1313	1000	1215	583	821
Of those selected with respect to mean activity (act; Figure 4): act_mvt_ > act_wait_ | act_wait_ > act_mvt_	758|223	1127|186	622|378	953|262	336|247	676|145
Selected neurons with >5 sp/s and >20 *m*-values for CV2 in both epochs (Figure 5 and Supplementary Figure S2)	944		884		481	
Selected neurons for phase-locking analysis with >30 spikes per analysis window (Figure 6)	1245	1408	1048	1167	848	1063

We found this result to be consistent for all trial types (see Supplementary Figure S2).

As evident from Figure 2, the time-resolved firing rate averaged across all neurons shows a clear increase during *movement* (see also Figure 3). However, in a minority of neurons (20%–30%) the firing rate decreased during *movement* as compared to the *wait* epoch (Figure 4A–C, right panels). We therefore explored whether the modulation of variability depends on these differential firing rate modulations. For that purpose we split the neurons into two subpopulations with respect to their firing rate profile during the two epochs. One subpopulation contained the majority of neurons with a firing rate that was higher during *movement* than during *wait* (80%, 70% and 70% in monkeys L, N and E, respectively; see Table 1), whereas the other subpopulation contained the remaining neurons with a higher firing rate during *wait* than during *movement*. We again found that the CV2 generally increases and FF generally decreases from *wait* to *movement* irrespective of the firing rates of the individual neurons (see Figure 5 for SG-HF and Supplementary Table S2 for data from all monkeys and trial types).

### Increased Spike Time Irregularity Coincides With Reduced Phase-Locking of Spikes to the LFP

Next, we explored why the CV2 is lower during *wait* than during *movement* (as shown in Figure 3). A possible reason for the more regular firing during *wait* than during *movement* may lie in the tendency of spikes to lock to LFP oscillations (Denker et al., 2007, 2011). The power of motor cortical LFP beta oscillations (15–35 Hz) strongly increases during an instructed delay, but is lowest during movement execution (Sanes and Donoghue, 1993; Pfurtscheller et al., 1996; Kilavik et al., 2012; for a review see Kilavik et al., 2013; see also Lebedev and Wise, 2000; for oscillatory spiking activity). We therefore hypothesized that a majority of neurons exhibit phase locking of their spikes to the beta oscillations during *wait* but not during *movement* where beta oscillations are vanishing. This would imply, as a consequence, that the spiking activity is more regular during *wait* than during *movement*.

Therefore, we analyzed LFP oscillatory activity in the beta range and related the spike times of each single neuron to the oscillation phase of the LFP signal. For each recording channel, we first averaged trial-by-trial the LFP signals recorded from all direct neighboring channels to exclude trivial signal correlations between the occurrence of spikes and the phase of the LFP oscillation induced by volume conductance effects (Katzner et al., 2009). Figure 6A shows a spectrogram obtained from such a LFP signal in a single session in monkey N, averaged across all trials of trial type SG-HF. A clear beta oscillation occurred during cue presentation and the subsequent delay period. The beta oscillation ceased during movement execution and only re-occurred around the reward (Rew) at the end of the trial. We next determined the spike occurrences of each neuron recorded on each electrode with respect to the beta phase of the LFP oscillation averaged across all neighboring electrodes (see “Materials and Methods” section). Figure 6B shows during five single trials the raw (averaged) LFP signals in gray, the beta-filtered signal (main beta frequency 18 Hz ± 5 Hz) in black, and the instantaneous oscillation phase in cyan. The spike times of one single neuron recorded on the center electrode are indicated by red dots.

We then determined for each neuron the phase values at its spike times in each of the two selected epochs, *wait* and *movement*, and calculated their phase distributions in circular space (see Figures 6C,D). To capture potential phase locking of the spike times, we evaluated the statistical significance (*p* < 0.05) of the non-uniformity of each distribution using the Rayleigh test (see Figures 6C,D, for the example data). Repeating this analysis for all neurons showed indeed a higher percentage of significantly phase-locked neurons during *wait* (black) than during *movement* (green), in all three monkeys (Figure 6E). Supplementary Figure S3 shows that the relative fractions of significantly phase-locked neurons during *wait* and *movement* which were almost the same in all trial types and for each monkey. Thus, phase-locking of spikes is prominent during *wait* but almost at chance level during *movement*.

### Context-Modulated Relationship of Spike Time Irregularity and Cross-Trial Spike Count Variability

For the final step of our analysis, we considered predictions from stochastic point process theory (see “Materials and Methods” section). For a given point process model, spiking irregularity (CV) and count variance (FF) are related in a unique manner. Under certain conditions, spike time irregularity and spike count variability are related as FF ≈ CV^2^ (Shadlen and Newsome, 1998; Stevens and Zador, 1998; Nawrot et al., 2007, 2008; Ditlevsen and Lansky, 2011). This assumes stationary conditions and is true for all renewal processes, where ISIs are independent and equally distributed, assumptions that are widely used as models for spiking activity (Perkel et al., 1967; Tuckwell, 1988; Chacron et al., 2001; Nawrot, 2010). The statistical equality of FF ≈ CV^2^ was confirmed in *in vitro* experiments that used stationary noise current injection to stimulate single cortical neurons (Stevens and Zador, 1998; Nawrot et al., 2003a,b, 2008).

**Figure 7:**
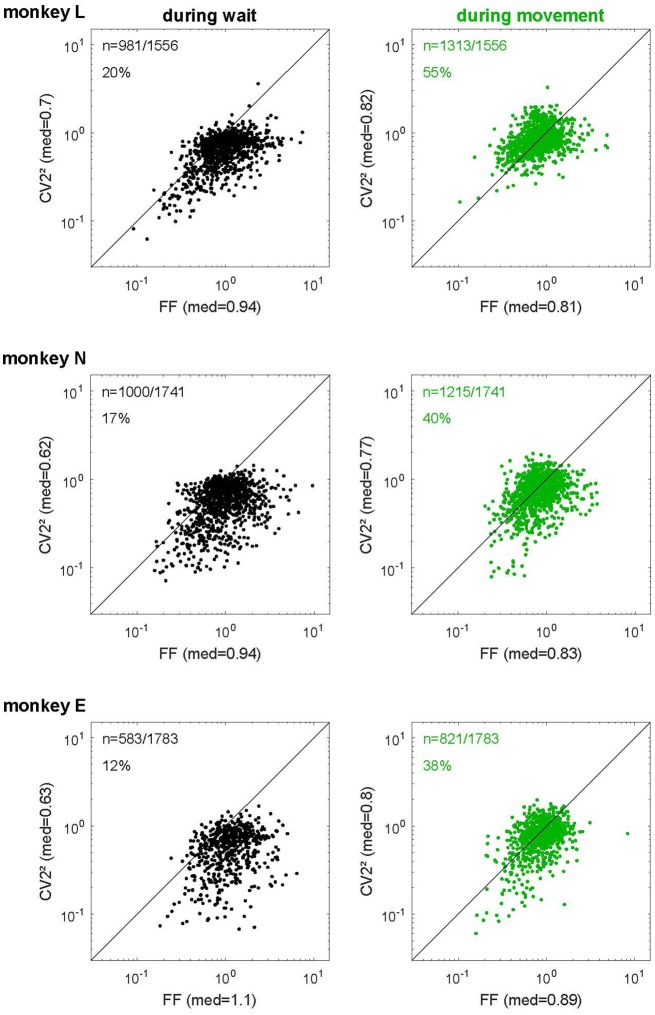
The relationship of spike time irregularity and spike count variability depends on the behavioral context. Log-log representation of scatter diagrams of the CV2^2^ vs. FF during *wait* (black, left) and *movement* (green, right) for each monkey in SG-HF. The medians of FF and CV2^2^ are indicated on each axis. In the left upper corner of each plot, the number of selected neurons and the percentage of neurons is indicated whose ratio FF/CV2^2^ was smaller than 1. These percentages as well as the ratio FF/CV2^2^ during all trial types and for all monkeys are shown in Supplementary Table S3.

We tested this relationship for the spiking data of our *in vivo* recordings and plotted the squared local measure, CV2^2^, against the FF. Figure 7 shows these scatter diagrams separately for each monkey and behavioral epoch. We found that the expectation for the hypothesis (see “Materials and Methods” section) was met in the *movement* epoch (Figure 7, right panels, in green), where all data points are close to the diagonal (on average FF/CV2^2^ = 0.96, 1.11 and 1.14 for monkeys L, N and E, respectively). The scattering around the diagonal can easily be explained by the variance of estimation, because we have only a limited number of trials per neuron (Nawrot, 2010). During *wait* (Figure 7, left panels, in black), however, there was a strong and systematic deviation from the diagonal, where FF was by far larger than CV2^2^ (on average FF/CV2^2^ = 1.43, 1.6 and 1.94 for monkeys L, N and E, respectively). In the *wait* epoch we found only a minority of single neurons (20%, 17% and 12% in monkeys L, N and E, respectively) for which FF was smaller than CV2^2^, i.e., FF/CV2^2^ < 1, whereas during *movement* context this held true for approximately half of the neurons (55%, 40% and 38% in monkeys L, N and E, respectively). Supplementary Table S3 shows that both the percentages of neurons with a smaller FF than CV2^2^ and the average ratios (median) of FF/CV2^2^ during *wait* and *movement* were very similar in all four trial types and all three monkeys. Our results indicate that during *wait* the theoretic prediction for renewal processes is clearly violated in a systematic manner whereas during *movement* the neurons match the renewal expectation FF ≈ CV^2^.

## Discussion

The present study provides new insights into the variability of single neuron spiking activity and their dependance on the behavioral context. In line with previous studies on trial-by-trial variability in motor cortex (Nawrot et al., 2003b; Churchland et al., 2006, 2010; Rickert et al., 2009), our results show that the FF decreases strongly from movement preparation (*wait)* to execution (*movement)*. The same task-related dynamics of across-trial count variability was confirmed for different cortical areas in a large meta-study (Churchland et al., 2010). In addition, we here report two new important findings. First, as opposed to the FF, the CV2 is on average significantly higher during *movement* than during *wait* (Figure 3), irrespective of the firing rate (Figure 5). Second, we found that the relation FF ≈ CV2^2^ for each neuron is fulfilled during *movement*, but not during *wait* where the FF was considerably larger than the CV2^2^ (Figure 7). Both results, detailed for one trial type (SG-HF) in the “Results” section, are consistent in all other trial types (see Supplementary Information, Supplementary Tables S1, S3).

### Context-Dependent Modulation of Spike Time Irregularity

Our data show that the CV2 increases from *wait* to* movement*. This is in agreement with Davies et al. (2006) who found during a PG task a considerable fraction of pyramidal tract neurons exhibiting more regular firing during hold than during movement execution. Similarly, Compte et al. (2003) showed in prefrontal neurons a modulation of CV2 as a function of task requirements, with a lower CV2 during eye fixation than during a more demanding mnemonic delay. Our results cannot be simply explained as an artifact of the firing rate modulation during *movement* as shown in ground truth simulation studies where similar rate modulations did not impact the CV2 (Ponce-Alvarez et al., 2010; Hamaguchi et al., 2011).

During *wait* our data show a low spike time irregularity as expressed by a low CV2. These results point to a strong link between the low CV2 and the high LFP beta power. Indeed, we find that during *wait* the spiking activity of a larger percentage of neurons is significantly phase-locked to LFP beta oscillations than during *movement* (see Figure 6E and Supplementary Figure S3). This is in agreement with the findings that high power LFP beta oscillations (15–35 Hz) were observed during *wait*, whereas during *movement* beta oscillations were lowest (see Figure 6A; Sanes and Donoghue, 1993; Pfurtscheller et al., 1996; Kilavik et al., 2012, 2013). Similar results have been described by Lebedev and Wise (2000) for the spiking activity exhibiting oscillatory auto-correlograms at the beginning of an instructed delay. Furthermore, Murthy and Fetz (1992, 1996) described that many motor cortical neurons tend to fire in phase with high amplitude LFP oscillations, but they did not provide any indication of regular firing in absence of LFP oscillations. Finally, beta power was also increased during active hold in a PG task compared to movement execution (Baker et al., 1997; Davies et al., 2006). Indeed, irregularity is shown to be lower in all periods of elevated beta oscillation, as described in the cited literature, than during periods with weak or no beta oscillations.

### Modulation of FF vs. CV2 Indicates Context-Dependent Change of the Cortical Network State

During *movement*, our data robustly obey the renewal prediction FF ≈ CV^2^. Theoretical considerations and *in vitro* experiments indicate that under stationary input conditions cortical neurons are close to the renewal prediction. This was confirmed in acute rat cortical slice preparations when single neurons were stimulated with stationary noise currents (Nowak et al., 1997; Stevens and Zador, 1998; Nawrot et al., 2003a,b, 2007, 2008). Increasing the input noise, for instance, through balancing excitation with inhibition, increases overall variability of the output spike trains albeit with a fixed relation of FF ≈ CV^2^. Furthermore, *in vivo* intracellular recordings in anesthetized rat somatosensory cortex showed that FF is equal or even slightly smaller than the CV^2^ (FF ≤ CV^2^) under stationary conditions (Nawrot et al., 2007). In visual cortex of behaving monkeys, Brostek et al. (2013) showed that spiking statistics of MSTd neurons, both during the presentation of moving visual stimuli and during ocular following, are in good agreement with the renewal prediction. Unfortunately the authors did not test the renewal prediction before stimulus presentation and/or eye movements, which would have been better suited for comparison with our results during *wait*.

During *wait* we found a strong deviation from the theoretic prediction for renewal processes with FF ≫ CV2^2^. This confirms preliminary results on a different and smaller data set of monkey motor cortical neurons analyzed during a period of movement preparation (Nawrot, 2010). There are two possible interpretations of this result (see “Materials and Methods” section). The spiking activity of individual neurons might deviate from the renewal statistics during *wait*. This interpretation is unsatisfying because the very same neurons show a renewal-like spiking statistics during *movement*. In addition, spiking statistics close to renewalty has also been demonstrated for cortical neurons *in vitro* that were stimulated with injection of stationary noise currents (see above). We clearly favor the alternative interpretation which is a violation of the stationarity assumption implying varying input conditions that change from trial to trial. We hypothesize that the large trial-by-trial variability *in vivo* during *wait* reflects a trial-to-trial change of the network state implying a trial-to-trial change of the single neuron input level. Indeed, mimicking non-stationary input conditions *in vitro* by noise current injections where the input noise level was mildly varied across trials resulted in a strongly increased FF ≫ CV^2^ (Nawrot et al., 2003c).

What could be the cause for the postulated change in network state across trials? Recent theoretical models provide phenomenological and mechanistic explanations for the observed modulation of the FF that support our interpretation of non-stationary conditions across trials. A simple phenomenological point-process model combined a renewal process with slow and moderate across-trial variation of the point process intensity (i.e., the single neuron firing rate) to account for ongoing network activity that is not task-related (Arieli et al., 1996). This resulted in a spontaneous spiking statistics where FF ≫ CV^2^. Adding a stereotyped task-related activation component that mimics activation during movement resulted in a reduction of FF such that FF ≈ CV^2^ (Nawrot, 2010). Recent studies of large-scale clustered spiking neural network models suggested an interesting mechanistic explanation for the task-related FF reduction. Neuron clusters formed by highly interlinked groups of excitatory neurons result in a network activity that cycles through different attractor states (Deco and Hugues, 2012; Litwin-Kumar and Doiron, 2012; Mazzucato et al., 2015). Combining excitatory and inhibitory neuron clusters further increases the robustness of the observed attractor dynamics. In spontaneous conditions this cluster cycling effectively leads to a permanent change in the network state. In neural network simulations with spiking neurons that mimic an experimental trial design this cluster dynamics results in a high single neuron spike count variability (high FF) before stimulation. However, when a particular cluster is stimulated, the network activity becomes focused on this cluster, while activity in non-stimulated clusters is suppressed through global inhibition. Trial-by-trial stimulation of the same cluster results in a strongly reduced trial-to-trial variability during stimulation in all neurons (Deco and Hugues, 2012; Litwin-Kumar and Doiron, 2012; Mazzucato et al., 2015). This model could reproduce the task-related FF dynamics observed *in vivo* (Churchland et al., 2010). However, these studies currently lack a detailed analysis of spiking irregularity that would allow for a thorough comparison with physiology.

An alternative model explanation is based on the self-inhibiting cellular mechanism of spike frequency adaptation (SFA) that is ubiquitous in spiking neurons including cortical neurons (Lundstrom et al., 2008). If model neurons in a cortical network with balanced excitation and inhibition are equipped with a SFA current, stimulation of an excitatory population leads to a strong stimulus-locked transitory reduction of FF that qualitatively and quantitatively fits the physiological *in vivo* observations (Farkhooi et al., 2013). Both suggested mechanisms - attractor dynamics at the network level and SFA at the cellular level - are likely to act in concert in order to produce the complex state and context-dependent FF dynamics observed in experimental data (Rickert et al., 2009; Churchland et al., 2010, 2011).

## Conclusion

Motor cortex is particularly well suited to explore how the dynamics of spiking activity is modulated by the behavioral context. Here we suggest that during *wait*, regularity expressed by a low CV2 is related to the increased power of LFP beta oscillations. Across-trial variability expressed by the FF, however, is high because in each trial the local network is in a different state implying a different amount of synaptic input and spike output of single neurons for each trial. This non-stationarity across trials was attributed to ongoing brain activity (Arieli et al., 1996). Indeed, motor cortical activity is modulated long before movement execution by processes such as visuomotor transformation (di Pellegrino and Wise, 1993; Riehle et al., 1997; Zhang et al., 1997) or movement preparation (Riehle, 2005; Confais et al., 2012). These specific trial-by-trial modulations are under the influence of upstream activity from sensory and associative areas and may be caused by attractor cycling in clustered cortical network models (Deco and Hugues, 2012; Litwin-Kumar and Doiron, 2012; Mazzucato et al., 2015). We speculate that FF is high while CV2^2^ is low during *wait* simply because we sample spiking of individual neurons across different network states. Thus the assumption of stationarity, which is implicit to the renewal assumption, is violated. Single neuron spiking might still represent renewal spiking albeit with a slightly different process rate in each trial (Nawrot, 2010). During *movement*, the local motor cortical network is recruited for performing the motor task. Single neurons receive strong task-related and stereotyped input. In effect, in each trial the neuronal population activity reliably occupies the same optimal subspace related to the desired movement (Churchland et al., 2006; Shenoy et al., 2013) and single neuron output shows a reduced trial-by-trial variability. The CV2, however, is enhanced due to the lack of phase locking, such that we find an agreement of FF and CV2.

## Author Contributions

TB designed the behavioral task. TB and AR performed surgery and recorded data. AR performed analysis. AR, TB, MN and SG discussed and interpreted the results, and contributed in writing the article. Experiments and analyses were performed at the INT.

## Conflict of Interest Statement

The authors declare that the research was conducted in the absence of any commercial or financial relationships that could be construed as a potential conflict of interest.
