# Supplementary material for: Behavioral Context Determines Network State and Variability Dynamics in Monkey Motor Cortex
Source: Front Neural Circuits. 2018 Jul 12;12:52. doi: 10.3389/fncir.2018.00052 (PMC6052126; doi:10.3389/fncir.2018.00052)
Supplement: Supplementary file 1 [file Data_Sheet_1.pdf]

Supplementary Information

Behavioral context determines network state and variability dynamics in monkey motor cortex

Alexa Riehle[1,2*], Thomas Brochier[1], Martin Nawrot[4,§], Sonja Grün[2,3,5,§]

[1] Institut de Neurosciences de la Timone (INT), Centre National de la Recherche Scientifique (CNRS) - Aix-Marseille Université (AMU), UMR7289, 13005 Marseille, France
[2] Institute of Neuroscience & Medicine (INM-6) & Institute for Advanced Simulation (IAS-6) & JARA Brain Institute I, Forschungszentrum Jülich, 52425 Jülich, Germany
[3] RIKEN Brain Science Institute, 2-1 Hirosawa Wako-Shi, Saitama 351-198, Japan
[4] Computational Systems Neuroscience, Institute of Zoology, University of Cologne, 50674 Cologne, Germany
[5] Theoretical Systems Neurobiology, RWTH Aachen University, 52062 Aachen, Germany

[§] *These authors contributed equally*

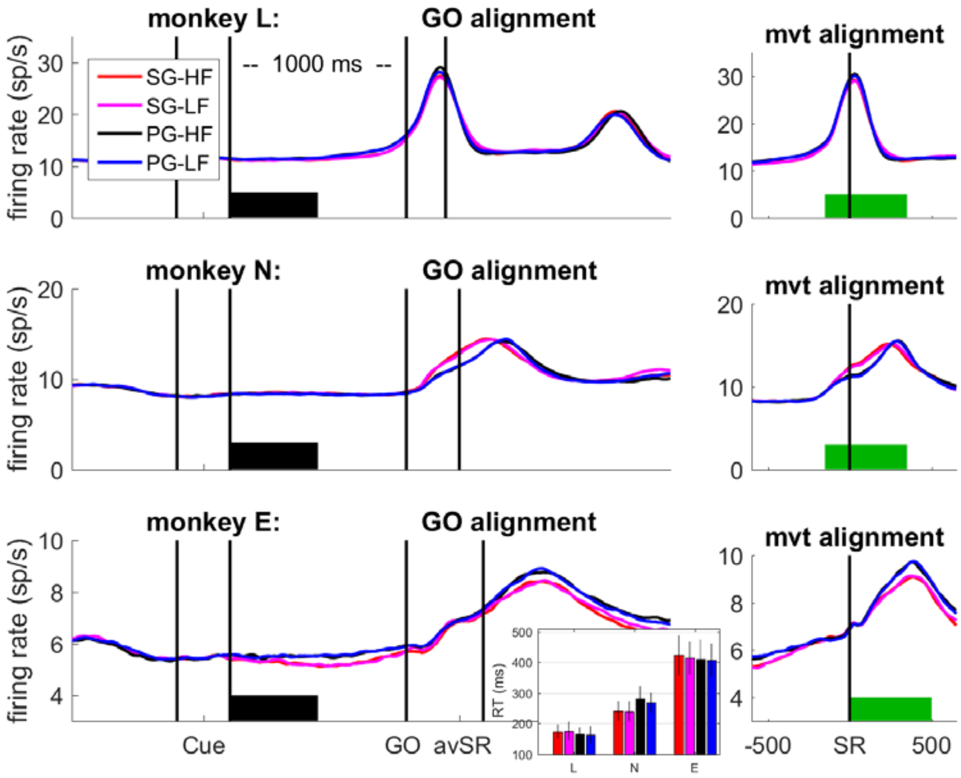

Figure S1. Task-related firing rates averaged across all selected neurons during correct trials for monkey L (top), monkey N (middle) and monkey E (bottom) during all trial types (see Fig. 2 for SG-HF). Data were aligned to the GO signal (left) and movement onset (switch realease, SR; right). The selected analysis windows of 500ms duration are indicated by a thick bar in black (*wait)* and green (*movement*). SG: side grip; PG: precision grip; HF: high force; LF: low force; Cue: preparatory cue; GO: Go signal; SR: switch release (movement onset). Number of neurons monkey L: n=1556, monkey N: n=1741, and monkey E: n=1783. It can clearly be seen that there were hardly no differences in firing rates between conditions for the entire data sets.

The inset in the lower panel shows the average reaction times (RT), i.e. the time between GO and SR, for each monkey and all four trial types.

**monkey**	**trial type**	**CV2 wait**	**CV2 mvt**	**FF wait**	**FF mvt**	**rate wait**	**rate mvt**
**L**	**SG-HF**	0.84	0.9	0.94	0.81	13.6	20.4
**L**	**SG-LF**	0.84	0.9	0.94	0.81	13.5	20.9
**L**	**PG-HF**	0.83	0.9	0.88	0.79	13.9	20.8
**L**	**PG-LF**	0.83	0.9	0.86	0.8	14	20.8
**N**	**SG-HF**	0.79	0.88	0.94	0.83	11.6	14.2
**N**	**SG-LF**	0.79	0.88	0.95	0.85	11.6	14.2
**N**	**PG-HF**	0.8	0.88	0.98	0.86	11.4	13.2
**N**	**PG-LF**	0.8	0.88	0.98	0.86	11.4	13.4
**E**	**SG-HF**	0.77	0.88	1	0.87	10.5	11.6
**E**	**SG-LF**	0.78	0.88	1	0.88	10.6	11.5
**E**	**PG-HF**	0.78	0.88	1.1	0.92	10.4	11.9
**E**	**PG-LF**	0.8	0.9	1.2	0.95	10.4	11.8

Table S1: CV2, FF, and firing rate during *wait* and *movement* during all trial types. All averages are medians. For each feature, differences between *wait* and *movement* are highly significant (Wilcoxon ranksum test; $p<10^{-4}$). Firing rate is in spikes/second.

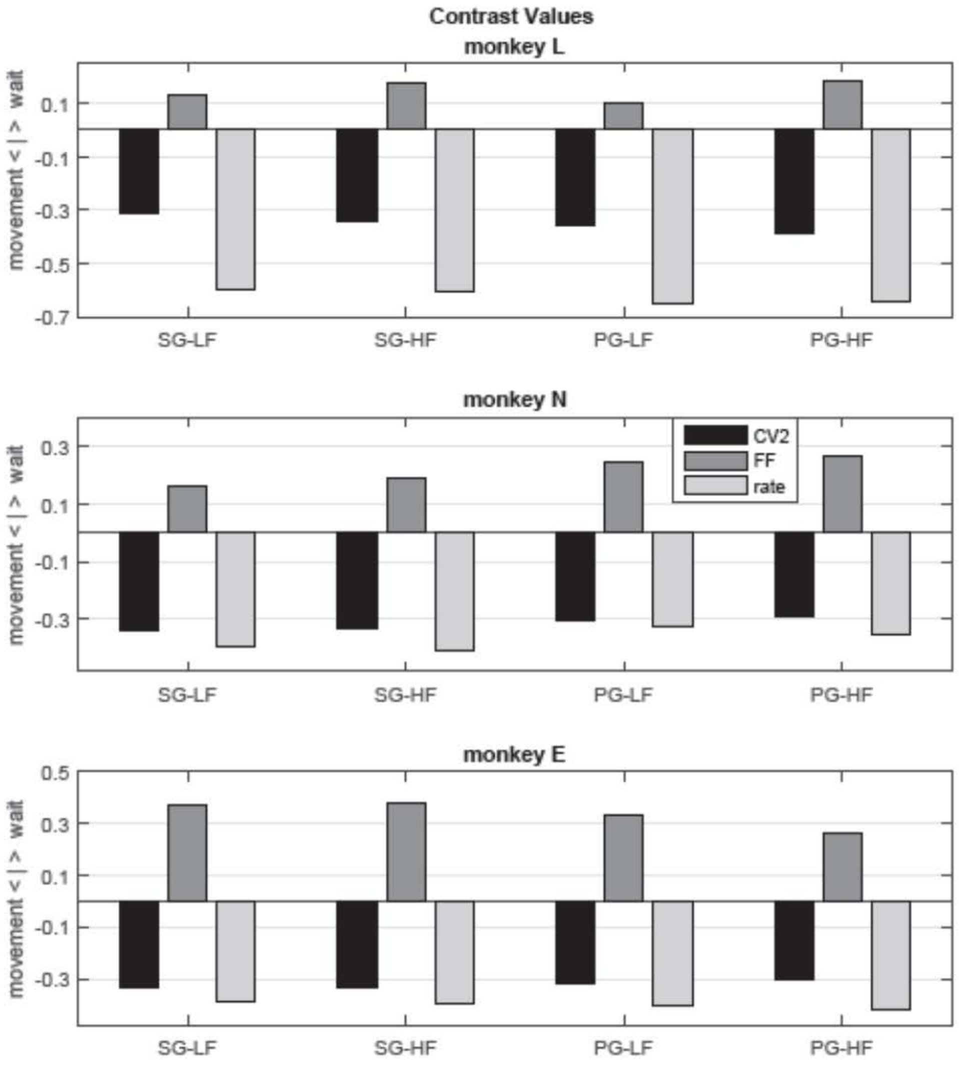

Figure S2: Contrast values for each measure (CV2, FF and firing rate) between *wait* and *movement*. Data obtained for each trial type and each monkey.

monkey	trial type	CV2 wait	sig	CV2 mvt	FF wait	sig	FF mvt	rate wait	sig	rate mvt
**L profile 1**	**SG-HF**	0.86	*	0.9	0.94	*	0.82	12.9	*	22.3
**L profile 1**	**SG-LF**	0.85	*	0.9	0.95	*	0.81	12.9	*	22.8
**L profile 1**	**PG-HF**	0.85	*	0.91	0.9	*	0.8	13.2	*	22.1
**L profile 1**	**PG-LF**	0.84	*	0.91	0.89	*	0.81	13.7	*	23
**L profile 2**	**SG-HF**	0.77	*	0.9	0.84	*	0.77	15.9	*	12.3
**L profile 2**	**SG-LF**	0.77	*	0.89	0.89	*	0.79	15.4	*	11.9
**L profile 2**	**PG-HF**	0.73	*	0.9	0.75	*	0.73	16.5	*	11.9
**L profile 2**	**PG-LF**	0.75	*	0.89	0.76		0.75	14.8	*	11.5
**N profile 1**	**SG-HF**	0.81	*	0.89	0.94	*	0.8	11.0	*	16.2
**N profile 1**	**SG-LF**	0.81	*	0.88	0.95	*	0.83	10.9	*	15.9
**N profile 1**	**PG-HF**	0.82	*	0.88	0.97	*	0.84	10.7	*	15.2
**N profile 1**	**PG-LF**	0.81	*	0.88	0.97	*	0.84	10.9	*	15.3
**N profile 2**	**SG-HF**	0.76	*	0.87	0.96		0.91	13.1	*	9.8
**N profile 2**	**SG-LF**	0.76	*	0.86	0.95		0.96	13.1	*	9.9
**N profile 2**	**PG-HF**	0.77	*	0.87	1.02	*	0.9	12.4	*	9.3
**N profile 2**	**PG-LF**	0.77	*	0.86	0.98		0.91	12.2	*	9.7
**E profile 1**	**SG-HF**	0.76	*	0.88	0.91	*	0.86	10.5	*	12.7
**E profile 1**	**SG-LF**	0.78	*	0.88	0.98		0.88	9.7	*	12.3
**E profile 1**	**PG-HF**	0.77	*	0.89	1.08	*	0.91	10.8	*	13.3
**E profile 1**	**PG-LF**	0.79	*	0.9	1.11	*	0.93	10.2	*	13.0
**E profile 2**	**SG-HF**	0.78	*	0.86	1.23	*	0.92	10.4	*	8.6
**E profile 2**	**SG-LF**	0.78	*	0.86	1.13	*	0.85	11.5	*	9.1
**E profile 2**	**PG-HF**	0.8	*	0.88	1.06		1.02	10.3	*	8.3
**E profile 2**	**PG-LF**	0.8	*	0.89	1.25	*	1.03	11.1	*	9.6

Table S2: CV2, FF and firing rate as a function of the behavioral context and firing rate profile (see also Fig. 5). We defined two types of firing rate profiles: the firing rate profile 1 had a firing rate during *movement* being larger than that during *wait* [act(mvt)>act(wait)], and *vice versa* the firing rate profile 2 had a firing during *wait* being larger than that during *movement* [act(wait)>act(mvt)].

Statistical significance of the differences each of the three measures between *wait* and *movement* is indicated by * in the row between the wait and movement columns ($p<0.05$; Wilcoxon ranksum test).

All CV2 differences between *wait* and *movement* were statistically significant and CV2 was always lower during *wait* than during *movement*. For rate profile 1, all FF differences were statistically significant and the FF was always higher during *wait* than during *movement*. However for rate profile 2, differences were not always significant, most likely due to the small number of neurons in these subpopulations (see Table 1). Note, that rate differences were by definition statistically significant, because they served as selection criterion.

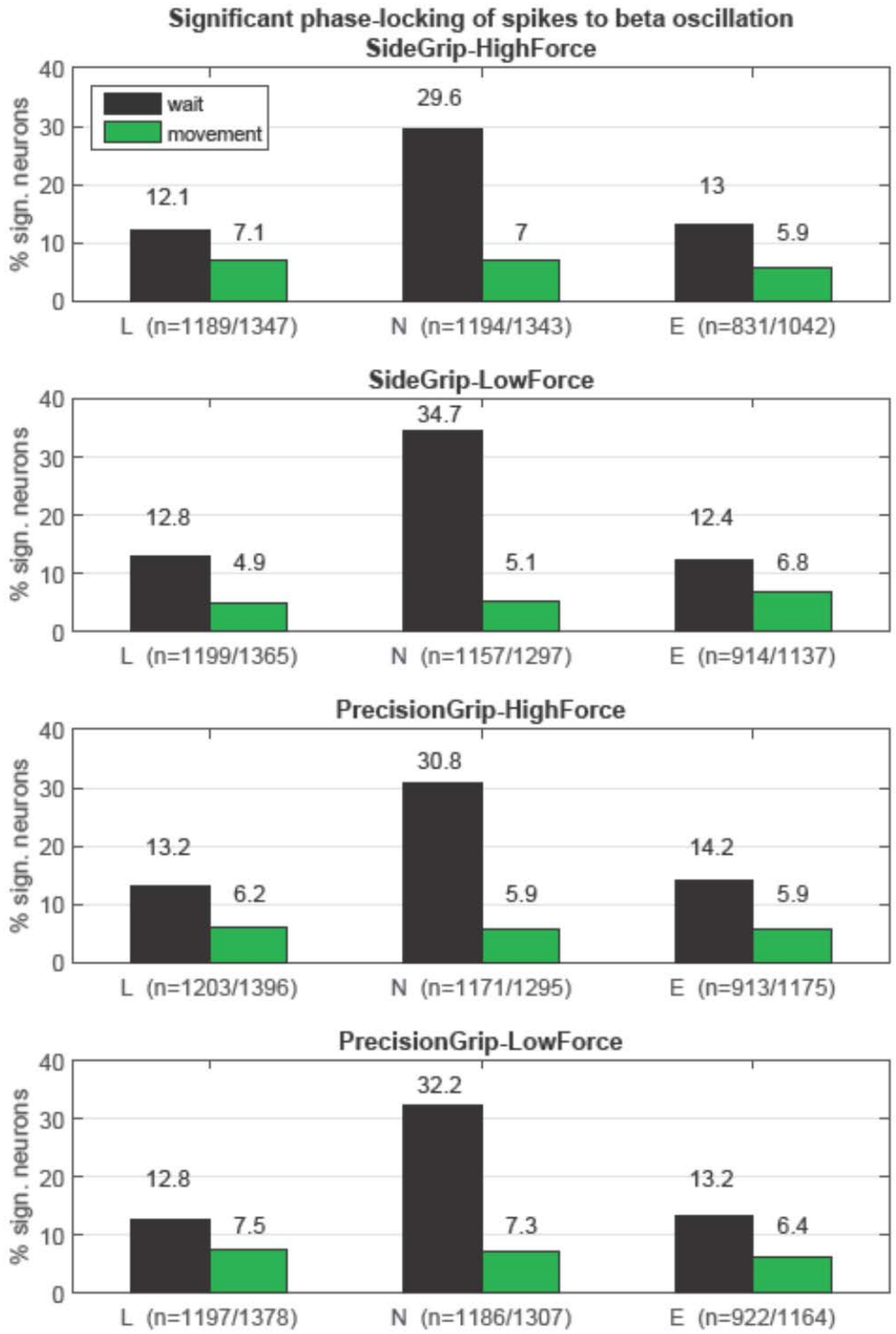

Figure S3: Percentages of neurons whose spike times are significantly ($p<0.05$) phase-locked to beta LFP oscillations in each epoch, trial type and monkey (L, N and E). Below each bar plot, the number of neurons analyzed during *wait*/*movement* are indicated in parenthesis.

**monkey**	**trial type**	**wait (%)**	**wait ($FF/CV2^2$)**	**movement (%)**	**mvt ($FF/CV2^2$)**
**L**	SG-HF	20	1.43	55	0.96
	SG-LF	20	1.46	52	0.97
	PG-HF	23	1.36	59	0.93
	PG-LF	22	1.38	54	0.96
**N**	SG-HF	17	1.6	40	1.11
	SG-LF	19	1.6	39	1.14
	PG-HF	15	1.65	36	1.15
	PG-LF	15	1.69	36	1.19
**E**	SG-HF	13	1.95	32	1.22
	SG-LF	13	1.92	34	1.21
	PG-HF	10	1.83	31	1.24
	PG-LF	8.2	2.05	35	1.22

Table S3: Test of renewal prediction (see Fig. 7). This table shows the percentages of neurons with a higher $CV2^2$ than FF during *wait* and during *movement*, for all trial types and all monkeys. This percentage is very low during *wait*, but approaches the 50% during *movement*. Furthermore, the median of the individual ratios $FF/CV2^2$ obtained for each neuron are shown. For renewal processes this ratio should be 1. The value of 1 is roughly obtained during *movement*, especially in monkey L, but not during *wait*, where the FF was by far larger than $CV2^2$. This table shows that the results during all trial types were very similar.
